# Plasma Proteomic Biomarkers in Alzheimer’s Disease and Cardiovascular Disease: A Longitudinal Study

**DOI:** 10.3390/ijms251910751

**Published:** 2024-10-06

**Authors:** Laurie A. Theeke, Ying Liu, Silas Wang, Xingguang Luo, R. Osvaldo Navia, Danqing Xiao, Chun Xu, Kesheng Wang, The Alzheimer and Disease Neuroimaging Initiative

**Affiliations:** 1 Department of Community of Acute and Chronic Care, School of Nursing, The George Washington University, Ashburn, VA 20147, USA; ltheeke@email.gwu.edu; 2 Department of Biostatistics and Epidemiology, College of Public Health, East Tennessee State University, Johnson City, TN 37614, USA; liuy09@etsu.edu; 3 Department of Statistics & Data Science, Dietrich College of Humanities and Social Sciences, Carnegie Mellon University, Pittsburgh, PA 15213, USA; silasw@andrew.cmu.edu; 4 Department of Psychiatry, Yale University School of Medicine, New Haven, CT 06516, USA; xingguang.luo@yale.edu; 5 Department of Medicine and Rockefeller Neuroscience Institute, West Virginia University, Morgantown, WV 26506, USA; ramiro.navia@hsc.wvu.edu; 6 Department of STEM, School of Arts and Sciences, Regis College, Weston, MA 02493, USA; danqing.xiao@regiscollege.edu; 7 Department of Health and Biomedical Sciences, College of Health Affairs, University of Texas Rio Grande Valley, Brownsville, TX 78520, USA; chun.xu@utrgv.edu; 8 Department of Biobehavioral Health & Nursing Science, College of Nursing, University of South Carolina, Columbia, 1601 Greene Street, Columbia, SC 29208, USA

**Keywords:** Alzheimer’s disease, mild cognitive impairment, cardiovascular diseases, proteomics, biomarkers, linear mixed model, *APOE*-ε4

## Abstract

The co-occurrence of Alzheimer’s disease (AD) and cardiovascular diseases (CVDs) in older adults highlights the necessity for the exploration of potential shared risk factors. A total of 566 adults were selected from the Alzheimer’s Disease Neuroimaging Initiative (ADNI) database, including 111 individuals with AD, 383 with mild cognitive impairment (MCI), and 410 with CVD. The multivariable linear mixed model (LMM) was used to investigate the associations of AD and CVD with longitudinal changes in 146 plasma proteomic biomarkers (measured at baseline and the 12-month follow-up). The LMM showed that 48 biomarkers were linked to AD and 46 to CVD (*p* < 0.05). Both AD and CVD were associated with longitudinal changes in 14 biomarkers (α1Micro, ApoH, β2M, BNP, complement C3, cystatin C, KIM1, NGAL, PPP, TIM1, THP, TFF3, TM, and VEGF), and both MCI and CVD were associated with 12 biomarkers (ApoD, AXL, BNP, Calcitonin, CD40, C-peptide, pM, PPP, THP, TNFR2, TTR, and VEGF), suggesting intricate connections between cognitive decline and cardiovascular health. Among these, the Tamm Horsfall Protein (THP) was associated with AD, MCI, CVD, and *APOE*-ε4. This study provides valuable insights into shared and distinct biological markers and mechanisms underlying AD and CVD.

## 1. Introduction

Alzheimer’s disease (AD), the leading cause of major neurocognitive disorders, affected 6.7 million Americans aged 65 and older in 2023 with projected increases to 13.8 million by 2060 [1]. Concurrently, cardiovascular diseases (CVDs) such as atherosclerosis, stroke, hypertension, myocardial infarction, coronary heart disease, and congestive heart failure are the foremost causes of disability and mortality globally for people aged 45 years and older [2,3]. CVDs impact 126.9 million adults in the United States (U.S.), a number that increases with age [4]. These conditions are particularly prevalent in the aging population, especially among adults aged 65 years and older, constituting an increasing public health problem [3,5,6,7].

Previous studies suggested that there is considerable overlap between AD and CVDs in terms of lifestyle risk factors, pathophysiology, and clinical presentations, but there might also be a direct causal association of CVDs and cardiovascular risk factors with AD and MCI [3,7,8,9]. For example, CVDs and risk factors are associated with an increased risk of AD and mild cognitive impairment (MCI), while AD and CVDs share similar genetic biomarkers and biochemical profiles, suggesting a complex interplay of genetic predispositions and environmental triggers [7,8]. Several genes have been linked to both AD and CVDs including apolipoprotein E (*APOE*), *MTHFR*, *HFE*, *FTO*, and *ARHGAP26* [8,10,11,12,13,14]. Most importantly, one study reported a shared *APOE-ε4* allele among several chronic health conditions, including AD, CVDs, and metabolic phenotypes [15]. A more recent study reported that ACE1 and several APO proteins were associated with AD, MCI, CVDs, and the *APOE-ε4* allele [16]. However, the mechanisms remain unclear. It has been proposed that certain genetic variants influence AD through multiple cardiovascular risk factors, [17] while some genes could interact with cardiovascular risk factors in the development of AD [18]. Additionally, an epigenetic/epigenomic influence in cardiovascular complications is also involved in the development of neurodegenerative disorders such as AD [19]. Therefore, there is still a gap in understanding the precise mechanistic pathways for the cause–effect and/or shared pathology between AD and CVDs [3].

Despite the established connections, there has been limited focus on the longitudinal changes in proteomic biomarkers in people suffering from both AD and CVD. Given the high prevalence of comorbid CVD in people with AD, exploring these biomarker changes is crucial for understanding the relationships between these conditions. This study aims to (1) examine the longitudinal changes in plasma proteomic biomarkers in adults who have been diagnosed with cognitive impairment (AD and MCI) and CVD while adjusting for covariates and (2) detect the overlapping biomarkers among AD, MCI, and CVD, thereby offering insights into potential shared pathophysiological mechanisms.

## 2. Results

### 2.1. Baseline Descriptive Statistics

At baseline, no significant differences were observed in age and education across groups defined by AD diagnostic status or CVD presence, indicating that these factors did not systematically vary among the groups studied (Table 1). Gender was not associated with AD but was associated with CVD. *APOE-ε4* was associated with AD diagnostic status but not with CVD.

### 2.2. Multivariable LMM Analysis

The LMM showed that AD and CVD were associated with longitudinal changes in 48 and 46 plasma proteomic biomarkers, respectively (*p* < 0.05, Table S1). Both AD and CVD were associated with longitudinal changes in fourteen proteomic biomarkers (α1Micro, ApoH, β2M, BNP, complement C3, cystatin C, KIM1, NGAL, PPP, TIM1, THP, TFF3, TM, and VEGF) (Table 2), of which four were also associated with MCI. Furthermore, MCI and CVD were associated with twelve proteomic biomarkers (ApoD, AXL, BNP, Calcitonin, CD40, C-peptide, pM, PPP, THP, TNFR2, TTR, and VEGF), with eight listed in Table 3. Moreover, the single biggest risk gene for sporadic AD, *APOE-ε4*, was associated with 22 proteomic biomarkers (Table S1).

The THP was associated with AD, MCI, CVD, and *APOE-ε4* (Table 2) and with age (*p* = 0.001). The multiple comparisons for the THP between AD diagnosis, CVD status, and *APOE* genotypes at baseline and the 12-month follow-up are illustrated in Table 4. There were significant differences between AD and CN, between CVD and non-CVD, and between *APOE*-*ε4*-1+ and *APOE*-*ε4*-0 at baseline and at the 12-month follow-up, as well as between MCI and CN at the 12-month follow-up.

### 2.3. Correlation Analysis and Variable Cluster Analysis

**Figure 1:**
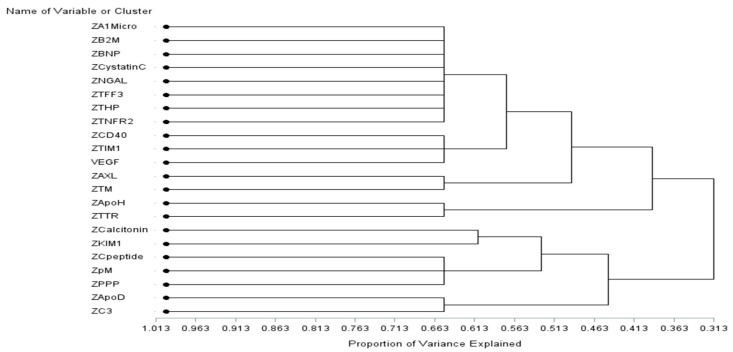
Oblique variable clustering analysis of 22 proteins.

Pearson and Spearman correlation analyses were conducted for the baseline data of 22 proteins which were associated with AD or MCI and CVD in LMM analysis (Table 2 and Table 3), and the coefficients are presented in Table S2. Based on variable cluster analysis, the twenty-two proteins were clustered into eight clusters (eight, three, two, two, one, one, three, and two variables for each cluster, Figure 1). Cluster 1 included eight proteins (α1Micro, β2M, BNP, cystatin C, NGAL, TFF3, THP, and TNFR2), while cluster 2 included three proteins (CD40, TIM1, and VEGF). AXL and TM were in cluster 3, and ApoH and TTR were in cluster 4. One protein (Calcitonin) was in cluster 5 and one protein (KIM1) was in cluster 6. Three proteins (C-peptide, pM, and PPP) were in cluster 7, and two proteins (ApoD and C3) were in cluster 8. About 65.1% of the total variation in the data could be accounted for by the eight clusters. Table S3 describes the variables in each cluster and the corresponding 1-R^2^ ratio values. Small values of the 1-R^2^ ratio indicate that the variable has a strong correlation with its own cluster and a weak correlation with the other clusters.

## 3. Discussion

This study underscores the complex relationships among AD, CVDs, and proteomic biomarkers in these conditions. There is established evidence that the pathway to the development of CVDs and AD includes regulating the transcription and translation of specific biomarkers, which in turn affects the structure and physiological function of resident cells in both the cardiovascular and central nervous system. After controlling for known potential confounding factors (age, gender, education, and *APOE-ε4* allele), the main findings of this study include that (1) AD and CVDs were associated with longitudinal changes in 48 and 46 proteomic biomarkers, respectively, and both AD and CVDs were associated with longitudinal changes in 14 proteomic biomarkers; (2) both MCI and CVDs were associated with longitudinal changes in 12 proteomic biomarkers; and (3) *APOE-ε4* was associated with 22 proteomic biomarkers. Interestingly, AD, CVDs, and *APOE-ε4* were associated with α1Micro, β2M, complement C3, cystatin C, and THP. Furthermore, MCI, CVDs, and *APOE-ε4* were associated with THP, Calcitonin, and TNFR2. Additionally, THP was associated with AD, MCI, CVD, and *APOE-ε4.*

### 3.1. Proteomic Biomarkers Associated with AD, CVDs, and APOE-ε4

Interestingly, both AD and CVDs were associated with longitudinal changes in 14 biomarkers (α1Micro, ApoH, β2M, BNP, complement C3, cystatin C, KIM1, NGAL, PPP, TIM1, THP, TFF3, TM, and VEGF); among them, five proteins (α1Micro, β2M, complement C3, cystatin C, and THP) were associated with *APOE-ε4* (Table 2).

Alpha-1-microglobulin (α1M) is a small plasma protein that plays a role in various physiological processes, including the regulation of the immune response and protection against oxidative stress. One previous study found that α1M may play a role in AD [20], and one study of α1M in acute kidney injury and cardiovascular events reported higher α1M levels in those with CKD, diabetes, hypertension, and heart failure [21]. Beta-2 microglobulin (*β2M*) is a low-molecular-weight protein that is a component of the major histocompatibility complex (MHC) class 1 molecules, present on the surface of nucleated cells. It has been primarily recognized for its role as a marker of immune system activity and renal function. It has been suggested as a potential biomarker for preclinical AD and might have varied functions throughout various stages of preclinical AD progression, possibly related to energy metabolism in the pathological mechanism of AD [22,23]. *β2M* is one of the genes that appear to mediate the association of diet with incident cardiovascular disease and all-cause mortality [24], and there are moderate positive associations between *β2M* levels and CVD events and mortality [25]. Recently, it was reported that β2M plays an active role in both brain injury and cognitive disorders in animal models [26].

Complement C3 is a central component of the complement system and plays a crucial role in inflammation and immunity and the pathophysiology of both CVDs and AD. It is well established that complement C3 is associated with atherosclerosis and CVDs [27,28]. The activation of C3 leads to the recruitment of inflammatory cells to the vessel wall, contributing to the formation and progression of atherosclerotic plaques. One recent study found that the complement C3 system is involved in the progression of atherosclerosis by vascular remodeling [29], while another study suggested that complement C3 is associated with a higher future risk of coronary heart disease [30]. In AD, complement C3 may reflect stage-associated biomarker changes in AD [31], and the presence of *APOE-ε4* has been associated with elevated C3 levels, resulting in elevated Alzheimer’s neurodegeneration, noting that amyloid has a regulating effect on the complement system and is linked to subsequent tau protein pathology [32]. C3 is one of the blood biomarkers for use in point-of-care diagnosis tools for AD [20], with one recent study confirming that C3 could be a biomarker in the early diagnosis of AD [33].

Regarding the cystatin C protein, cystatin C is known to have neuroprotective properties. It is represented in neurodegenerative diseases [34] and is a therapeutic candidate that can potentially prevent brain damage and neurodegeneration [35]. One recent study provided evidence for the use of saliva samples to source biomarkers (including cystatin C) for the early detection of cognitive impairment in individuals on the AD continuum and potentially other neurodegenerative diseases [36]. However, the role of cystatin C in CVD showed inconsistent results. Previous studies revealed that cystatin C is historically linked to its utility as a marker of renal function which is closely linked to cardiovascular health. It can be viewed as an alternative predictor of complications for CVDs [37], and cystatin C could be promising as a biomarker in the diagnosis of coronary artery disease, aneurysm, adiposity, peripheral arterial disease, and coronary artery calcification [38]. A meta-analysis found that there is a significant association between high levels of cystatin C and the development of cardiovascular events or mortality in individuals with normal renal function [39]. One recent study also found that cystatin C independently predicted major cardiovascular events, the development of chronic kidney disease, and cardiovascular and all-cause mortality. The prediction of long-term mortality was independent of the improved estimation of GFR [40]. However, Mendelian randomization analyses did not support a causal role of cystatin C in the etiology of CVDs, and as such, therapeutics targeting lowering circulating cystatin C are unlikely to be effective in preventing CVDs [41].

### 3.2. Proteomic Biomarkers Associated with MCI, CVDs, and APOE-ε4

The present study further found that MCI and CVDs were associated with 12 biomarkers (ApoD, AXL, BNP, Calcitonin, CD40, C-peptide, pM, PPP, THP, TNFR2, TTR, and VEGF), while MCI, CVDs, and the *APOE-ε4* allele were associated with THP, Calcitonin, and TNFR2 (Table 2 and Table 3).

The current findings of cytokines (TNF-α, TNFR2, and VEGF) associated with CVDs are congruent with the results of previous studies for TNFR2 in myocardial infarction in the Swedish population [42] and TNF-α [43]. The findings of this study are important in understanding how VEGF contributes to cardiovascular illness. An alteration in VEGF has been associated with several diseases (e.g., kidney, hypertension, cancer, or diabetes), but this study was the first report showing that VEGF is increased in CVDs. VEGF represents inflammatory processes that were present in several tissues including the heart and brain. These CVD-associated biomarkers could be used to inform clinical applications, such as pharmacotherapies targeting neutralizing inflammatory biomarkers which have displayed potential for effectiveness toward treating CVDs, such as atherosclerosis, although contradictory findings indicate that there is still a need for designing more precise therapy [44]. VEGF was positively associated with AD, MCI, and CVDs in our study. Thus, the biomarkers shared by AD, MCI, and CVDs in the current study can be used to further identify shared pathophysiology for AD, MCI, and CVDs. VEGF, as an intracellular player together with other cytokines, may determine vascular risk factors and their contribution to cognitive impairment and the development of CVDs and AD. Understanding the complex interactions between VEGF and other signaling pathways could tell us how molecular discovery can inform future drug development and clinical trial design [45].

TNFR2 is one of two receptors of the cytokines and has proinflammatory effects. A recent study suggested that stimulating TNFR2 has the potential to strongly modulate the balance between effector T cells and Treg cells that may impact disease in both positive and negative manners [46]. Previous studies have shown that TNFR2 has a neuroprotective function. For example, a study in human post-mortem AD brain tissues demonstrated that TNFR2 levels were decreased [47], while individuals with low levels of TNFR2 were more likely to develop AD [48]. The neuroprotective role of TNFR2 signaling has been reported in a review article [49]. Furthermore, both animal model and human studies have suggested TNFR2 as a therapeutic target [50,51,52]. The present study revealed that TNFR2 was positively associated with CVD but negatively associated with MCI, whereas no significant association was found with AD. These findings suggest a differential role of TNFR2 in CVDs versus neurocognitive function and the complex pathogenesis mechanism of AD, MCI, and CVDs.

### 3.3. Proteomic Biomarkers Associated with AD, MCI, CVDs, and APOE-ε4

We found that AD, MCI, and CVDs were associated with BNP, PPP, THP, and VEGF, while THP was also associated with the *APOE-ε4* allele (Table 2).

The THP, also known as uromodulin, is associated with kidney function along with graft survival, CVDs, glucose metabolism, end-stage renal disease, systemic inter-organ signaling, and overall mortality [53,54]. For example, one previous study found that higher levels of urinary uromodulin were associated with a lower risk of eGFR decline, a lower risk of incident chronic kidney disease (CKD), and a lower risk of mortality in the aged population [55]. One recent study demonstrated that higher serum uromodulin levels were associated with lower mortality and major adverse cardiovascular events in a white CKD population from Germany [56]. A recent study reported that they were able to predict, with accuracy, the risk of a person with MCI progressing to dementia due to AD in a time period of up to four years by using a machine learning-based panel composed of 12 plasma proteins (ApoB, Calcitonin, C-peptide, CRP, IGFBP-2, Interleukin-3, Interleukin-8, PARC, Serotransferrin, THP, TLSP 1-309, and TN-C) that included THP [57].

### 3.4. Relationship among Shared Proteomic Biomarkers

The present study found that both AD and CVDs were associated with fourteen proteomic biomarkers, and both MCI and CVDs were associated with twelve proteomic biomarkers; among them, four biomarkers were shared by AD, MCI, and CVDs (Table 2 and Table 3). Based on the 22 shared proteins, correlation analyses and variable cluster analysis were conducted (Tables S2 and S3 and Figure 1). The twenty-two proteins were clustered into eight clusters. Proteins within a cluster revealed strong correlations with each other, which implies similar pathways. However, further pathway analysis is required to validate the results.

AD is an age-related neurodegenerative disorder which is characterized by the progressive accumulation in the brain parenchyma of β-amyloid A(β) plaques (Aβ peptides) and neurofibrillary tangles (tau protein), and the most widely used biomarkers for AD include the *APOE* genotype, CSF Aβ42, pTau and tTau, and findings from imaging setups such as MRI and PET together with a battery of cognition tests [34,58,59,60]. Previous studies have shown that the levels of Aβ42 are fully decreased at least 5 to 10 years before the conversion of MCI to AD dementia [61], the abnormal accumulation of Aβ can start decades before the dementia stage [62], and the first Aβ plaques occur at least 10 years, and probably 20–30 years, before the first symptoms [63]. Previous studies have also shown that amyloid-Aβ is increased in atherosclerotic arteries, indicating that amyloid-Aβ is involved in cardiovascular disease [64,65,66]. More recently, a study reported that ACE1 and several APO proteins are associated with AD, MCI, CVDs, and the *APOE*-ε4 allele [16]. The shared proteins among AD, MCI, and CVDs support previous studies that suggested that AD, MCI, and CVDs shared similar genetic biomarkers and biochemical profiles [7,8,10,11,12,13,14,16]. Furthermore, among the twenty-two shared proteins, seven biomarkers were associated with *APOE-ε4* (Table 2 and Table 3), providing additional evidence that the APOE-ε4 allele plays an important role in AD, MCI, CVDs, and metabolic phenotypes [15,16].

### 3.5. Strengths and Limitations

We are aware of the strengths of the current study. First, we have taken into account potential confounding factors in our analysis since the presence of certain confounding factors, such as variations in age, sex, and the *APOE-ε4* allele, may contribute to variations in findings [43] and false results. Second, we used longitudinal analysis, which is particularly useful for evaluating the relationship between risk factors and the development of diseases, such as AD and CVDs.

We acknowledge some limitations of the current study. First, we were unable to model the interactions between genetic, environmental, and direct effects nor document that genetic and environmental factors are considered risk factors for CVDs and AD since both diseases are considered complex and multifactorial. Second, the modest sample size in the current study may result in false positive or negative findings; thus, a future study with larger sample sizes is needed for accurate type-1 error rate control. To confirm the utility of these biomarkers, it is crucial to validate them in studies with larger samples. Third, in the future, further studies are needed to explore the inflammatory responses in the central nervous system of patients with AD and CVD to gain a better understanding of the disease’s etiology and risk factors.

## 4. Materials and Methods

### 4.1. Dataset

To achieve the aims of this study, data were analyzed from the Alzheimer’s Disease Neuroimaging Initiative (ADNI) database (adni.loni.usc.edu) (accessed on 26 October 2022). Initiated in 2003, the ADNI is an ongoing, longitudinal, multicenter study representing a collaborative effort as a public–private partnership, led by Principal Investigator Michael W. Weiner, MD. The primary goal of the ADNI is to test whether serial magnetic resonance imaging (MRI), positron emission tomography (PET), other biological markers, and clinical and neuropsychological assessment can be combined to measure the progression of MCI and early AD. The ADNI provides services in the U.S. and Canada. The use of ADNI data to perform a secondary data analysis is exempt from the Institutional Review Board.

### 4.2. Measures

The demographic variables included in this study were age, gender, and educational levels (https://adni.bitbucket.io/reference/ptdemog.html, accessed on 26 October 2022). Gender was a dichotomous variable that was self-reported as either male or female. Age and education were continuous variables expressed in total years. CVDs were identified using the MedHist data within the ADNI (https://adni.bitbucket.io/reference/medhist.html, accessed on 26 October 2022) with participants classified based on their history of CVDs, defined as either yes (with history) or no (without history). Data on *APOE-ε4* genotypes were extracted from the ADNI database (https://adni.loni.usc.edu/updated-apoe-results-2, accessed on 26 October 2022). *APOE* genotyping was performed on DNA samples obtained from blood samples. *APOE-ε4* carriers were defined as individuals with at least one ε4 allele (ε4/ε4 designated as *APOE-ε4*-2, ε4/ε3 or ε4/ε2 as *APOE-ε4*-1+), while non-carriers were defined as individuals with no ε4 allele (*APOE-ε4*-0) (Table 1).

Plasma samples were collected from a subset of these participants 12 months after the baseline assessment. Plasma samples were assayed for 190 analytes using the “Human DiscoveryMAP”, developed on the Luminex xMAP platform by Rules-Based Medicine. A total of 146 plasma proteomic biomarkers passed the strict ADNI quality control from a subset of the ADNI-1 dataset known as the “Biomarkers Consortium Plasma Proteomics Project RBM multiplex data” (https://adni.bitbucket.io/reference/rbmqc.html, accessed on 26 October 2022) [67]. More details about the Biomarkers Consortium Project “Use of Targeted Multiplex Proteomic Strategies to Identify Plasma-Based Biomarkers in Alzheimer’s Disease” are listed at https://adni.loni.usc.edu/wp-content/uploads/2010/12/BC-Plasma-Proteomics-Analysis-Plan.pdf, accessed on 26 October 2022. There were 566 adults measured for plasma proteomic biomarkers at baseline in the ADNI-1 dataset. This study included the demographics, AD diagnosis, medical history information, *APOE* genotypes, and protein data, and there were 111 individuals with AD, 383 with mild cognitive impairment (MCI), and 410 with CVD.

### 4.3. Statistical Methods

The categorical variables were depicted using raw counts along with the corresponding proportions, while continuous variables were presented as means ± standard deviations (SDs). The Chi-square test was used to examine the differences in categorical variables for both AD diagnoses and CVDs. For continuous variables, differences among groups based on AD and CVD diagnoses were evaluated using the one-way Analysis of Variance (ANOVA). Given the potential for a skewed distribution among the protein data, Z scores were calculated for each protein to standardize the data, using the mean and standard deviation.

A multivariable linear mixed model (LMM) was used to explore the impact of AD, CVDs, and the *APOE-ε4* allele on the longitudinal changes in 146 proteins (from baseline to 12 months). The model was adjusted for age, gender, and education.

(1) $$Y_{\mathrm{it}}=\mu_{t}+\beta_{i}x_{it}+\gamma z_{i}+\alpha_{i}+\varepsilon_{it}\;\;i=1,\ldots ,n;\;t=1,\ldots ,T$$

where Y*_it_* is the value of the outcome for individual *_i_* at follow-up time *_t_*, *μ_t_* is an intercept varying with time, *x_it_* is a vector of time-varying variables such as the follow-up visit, *z_i_* is a vector of time-invariant variables such as gender and education, *α_i_* denotes the random effects where each has a normal distribution with a mean of 0 and constant variance, and β are fixed effects. *ε_ij_* is a random distribution term. *i* = 1,…, I_j_ is the level-1 individual *i* indicator, and *t* = 1,…,T is the level-2 indicator, such as the follow-up visit.

Both Pearson and Spearman correlation analyses were performed to test the associations among AD- or MCI- and CVD-associated proteins using the Z scores. To further test the relationship among AD -or MCI- and CVD-associated proteins, the variable cluster procedure (VARCLUS) in SAS 9.4 (SAS Institute, Cary, NC, USA) was used to identify clusters of variables that exhibited strong correlations within each cluster and weak correlations with variables in other clusters, offering insight into the potential underlying patterns and relationships among the proteins. A good fit for each item was indicated by higher squared correlation (R^2^) values within its own cluster, low R^2^ values with the next closest cluster, and low 1 − R^2^ ratios (the ratio of 1 − R^2^ for a variable’s own cluster to 1 − R^2^ for its nearest cluster) [68,69].

(2) $$1-R^{2}\;ratio=\frac{1-R^{2}\;own\;cluster}{1-R^{2}\;next\;closest\;cluster}$$

All statistical analyses were performed using SAS (version 9.4). A *p* < 0.05 was considered statistically significant.

## 5. Conclusions

The findings from our study align with prior experimental and observational studies on the connection between biomarkers and two diseases (AD and/or CVD) after controlling for several potential confounding factors. Moreover, we observed a correlation between certain proteins and the *APOE-ε4* allele. Together, these biomarkers hold significant potential for early detection, prognostication, and perhaps even the development of targeted treatment therapies for AD and CVD. This potential can only be realized through the confirmation and validation of these findings in studies with larger sample sizes and well-defined traits/diseases. The further testing of additional biomarkers could contribute to the creation of a predictive scoring system, offering guidance for timely treatment interventions and future meta-analysis.

## Figures and Tables

**Table 1:** Descriptive statistics at baseline.

Variable	AD	MCI	CN	F/χ^2^, *p*	CVD	Non-CVD	*t*/χ^2^, *p*
Age (mean ± SD)	74.8 ± 8.1	74.8 ± 7.7	75.1 ± 5.8	0.05, 0.9527	75.2 ± 7.2	73.9 ± 7.8	3.60, 0.0584
Gender							
Male	65	248	30	4.39, 0.1113	258	85	4.38, 0.0364 *
Female	46	135	28		140	69	
Education (mean ± SD)	15.1 ± 3.2	15.6 ± 3.0	15.7 ± 2.8	1.31, 0.2699	15.4 ± 3.1	15.8 ± 2.8	1.70, 0.1930
*APOE- ε4*							
0	36	178	53	54.80, <0.0001 ***	187	80	1.10, 0.2953
1+	75	205	5		211	74	

Abbreviations: CN: cognitive normal; MCI: mild cognitive impairment; AD: Alzheimer’s disease; CVD: cardiovascular disease; SD: standard deviation. *p* value is based on Chi-square test or F test/*t* test in ANOVA. * *p* < 0.05; *** *p* < 0.0001.

**Table 2:** Multivariable linear mixed model for AD and CVD shared proteins.

Variable	AD vs. CN (*t*, *p*)	MCI vs. CN (*t*, *p*)	CVD vs. Non-CVD (*t*, *p*)	*APOE-ε4-1+* vs. 0 (*t*, *p*)	12 Months (*t*, *p*)
A1Micro	2.40, 0.0168 *	1.80, 0.0720	4.68, <0.0001 ***	−2.01, 0.0454 *	−5.20, <0.0001 ***
ApoH	2.20, 0.0279 *	−1.55, 0.1225	2.90, 0.0039 **	−0.54, 0.5874	−15.40, <0.0001 ***
β2M	3.10, 0.0020 **	0.28, 0.7813	4.52, <0.0001 ***	−2.61, 0.0092 **	−0.97, 0.3314
BNP	5.53, <0.0001 ***	4.94, <0.0001 ***	3.82, 0.0001 **	−0.40, 0.6866	3.728, 0.0011 **
Complement C3	2.66, 0.0080 **	−1.44, 0.1504	2.07, 0.0391 *	−2.30, 0.0221 *	2.94, 0.0034 **
Cystatin C	2.31, 0.0213 *	−1.24, 0.2157	5.78, <0.0001 ***	−2.49, 0.0130 *	−1.65, 0.1001
KIM1	−3.06, 0.0023 **	0.74, 0.4617	3.12, 0.0019 **	0.01, 0.9900	−5.47, <0.0001 ***
NGAL	2.83, 0.0048 **	0.60, 0.5465	2.53, 0.0117 *	−1.61 0.1080	−3.94, <0.0001 ***
PPP	3.53, 0.0005 **	3.08, 0.0022 **	2.15, 0.0322 *	1.31, 0.1904	2.77, 0.0059 **
TFF3	2.84, 0.0046 **	0.30, 0.7653	4.30, <0.0001 ***	−1.37, 0.1706	4.09, <0.0001 ***
THP	−3.51, 0.0005 **	−2.91, 0.0038 **	−4.10, <0.0001 ***	2.90, 0.0038 **	−9.98, <0.0001 ***
TIM1	3.29, 0.0011 **	0.68, 0.4999	2.14, 0.0328 *	−1.51, 0.1321	0.72, 0.4744
TM	2.14, 0.0324 *	0.90, 0.3665	4.19, <0.0001 ***	−0.74, 0.4626	−1.46, 0.1455
VEGF	3.63, 0.0003 **	2.40, 0.0169 *	4.13, <0.0001 ***	−1.70, 0.0895	1.92, 0.0553

Abbreviations: CN: cognitive normal; MCI: mild cognitive impairment; AD: Alzheimer’s disease; CVD: cardiovascular disease; LMM: linear mixed model; *t*-value is based on multivariable LMM adjusted for age, gender, education, and *APOE*-*ε4*. *p* value is based on multivariable LMM. * *p* < 0.05; ** *p* < 0.01; *** *p* < 0.0001.

**Table 3:** Multivariable linear mixed model for MCI and CVD shared proteins.

Variable	AD vs. CN (*t*, *p*)	MCI vs. CN (*t*, *p*)	CVD vs. non-CVD (*t*, *p*)	*APOE-ε4-1+* vs. 0 (*t*, *p*)	12 Months (*t*, *p*)
ApoD	−1.84, 0.0670	−3.12, 0.0019 **	−2.00, 0.0456 *	−0.92, 0.3576	−0.78, 0.4374
AXL	1.62, 0.1067	2.64, 0.0084 **	2.28, 0.0233 *	−0.25, 0.8027	−5.62, <0.0001 ***
Calcitonin	1.86, 0.0633	2.75, 0.0061 **	2.17, 0.0302 *	2.51, 0.0124 *	−2.34, 0.0197 *
CD40	1.15, 0.2498	−2.61, 0.0093 **	−0.93, 0.3543	−0.42, 0.6278	2.97, 0.0032 **
C-peptide	1.14, 0.2558	2.03, 0.0429 *	4.77, <0.0001 ***	0.22, 0.8281	1.05, 0.2947
pM	0.56, 0.5784	2.10, 0.0365 *	3.63, 0.0003 **	−1.15, 0.2525	1.95, 0.0517
TNFR2	0.64, 0.5208	−2.07, 0.0385 *	3.96, <0.0001 ***	−2.38, 0.0175 *	−3.43, 0.0007 **
TTR	−1.42, 0.1562	−3.69, 0.0002 **	2.68, 0.0075 **	1.10, 0.2701	−10.65, <0.0001 ***

Abbreviations: CN: cognitive normal; MCI: mild cognitive impairment; AD: Alzheimer’s disease; CVD: cardiovascular disease; LMM: linear mixed model; *t*-value is based on multivariable LMM adjusted for age, gender, education, and *APOE*-*ε4*. *p* value is based on multivariable LMM. * *p* < 0.05; ** *p* < 0.01; *** *p* < 0.0001.

**Table 4:** Multiple comparison for the THP in linear mixed model analysis.

Comparison	Visit	Difference ± SE	*t*, *p*
AD vs. CN	Baseline	−0.52 ± 0.16	−3.16, 0.0048 **
	12 months	−0.60 ± 0.16	−3.66, 0.0008 **
MCI vs. CN	Baseline	−0.31 ± 0.14	−2.22, 0.0695
	12 months	−0.50 ± 0.14	−3.53, 0.0013 **
AD vs. MCI	Baseline	−0.20 ± 0.10	−1.97, 0.1219
	12 months	−0.10 ± 0.11	−0.99, 0.5863
CVD vs. non-CVD	Baseline	−0.39 ± 0.09	−4.32, <0.0001 ***
	12 months	−0.33 ± 0.09	−3.55, 0.0004 **
*APOE-ε4-1+* vs. *0*	Baseline	0.24 ± 0.09	2.73, 0.0066 **
	12 months	0.26 ± 0.09	2.94, 0.0034 **

Abbreviations: CN: cognitive normal; MCI: mild cognitive impairment; AD: Alzheimer’s disease; CVD: cardiovascular disease; LMM: linear mixed model; t-value is based on multivariable LMM adjusted for age, gender, education, and *APOE*-*ε4*. *p* value is based on multivariable LMM. SE = standard error; *p* value is Tukey–Kramer Test ** *p* < 0.01; *** *p* < 0.0001.

## Data Availability

The data used in the preparation of this article were obtained from the Alzheimer’s Disease Neuroimaging Initiative (ADNI) database (adni.loni.usc.edu).

## Supplementary Materials

The following supporting information can be downloaded at https://www.mdpi.com/article/10.3390/ijms251910751/s1.

## References

[1] (2023). 2023 Alzheimer’s Disease Facts and Figures. Alzheimer’s Dement.

[2] Roth G.A., Mensah G.A., Johnson C.O., Addolorato G., Ammirati E., Baddour L.M., Barengo N.C., Beaton A.Z., Benjamin E.J., Benziger C.P. (2020). Global Burden of Cardiovascular Diseases and Risk Factors, 1990–2019: Update From the GBD 2019 Study. J Am Coll Cardiol.

[3] Waigi E.W., Webb R.C., Moss M.A., Uline M.J., McCarthy C.G., Wenceslau C.F. (2023). Soluble and Insoluble Protein Aggregates, Endoplasmic Reticulum Stress, and Vascular Dysfunction in Alzheimer’s Disease and Cardiovascular Diseases. GeroScience.

[4] Virani S.S., Alonso A., Aparicio H.J., Benjamin E.J., Bittencourt M.S., Callaway C.W., Carson A.P., Chamberlain A.M., Cheng S., Delling F.N. (2021). Heart Disease and Stroke Statistics—2021 Update: A Report From the American Heart Association. Circulation.

[5] Carter C.J., France J., Crean S., Singhrao S.K. (2017). The Porphyromonas Gingivalis/Host Interactome Shows Enrichment in GWASdb Genes Related to Alzheimer’s Disease, Diabetes and Cardiovascular Diseases. Front. Aging Neurosci..

[6] Gong S., Su B.B., Tovar H., Mao C., Gonzalez V., Liu Y., Lu Y., Wang K.-S., Xu C. (2018). Polymorphisms Within RYR3 Gene Are Associated With Risk and Age at Onset of Hypertension, Diabetes, and Alzheimer’s Disease. Am. J. Hypertens..

[7] Tini G., Scagliola R., Monacelli F., La Malfa G., Porto I., Brunelli C., Rosa G.M. (2020). Alzheimer’s Disease and Cardiovascular Disease: A Particular Association. Cardiol. Res. Pract..

[8] Mur J., McCartney D.L., Walker R.M., Campbell A., Bermingham M.L., Morris S.W., Porteous D.J., McIntosh A.M., Deary I.J., Evans K.L. (2020). DNA Methylation in APOE: The Relationship with Alzheimer’s and with Cardiovascular Health. AD Transl. Res. Clin. Interv..

[9] De Bruijn R.F., Ikram M.A. (2014). Cardiovascular Risk Factors and Future Risk of Alzheimer’s Disease. BMC Med..

[10] Eriksson U.K., Bennet A.M., Gatz M., Dickman P.W., Pedersen N.L. (2010). Nonstroke Cardiovascular Disease and Risk of Alzheimer Disease and Dementia. Alzheimer Dis. Assoc. Disord..

[11] Kotze M.J., Van Rensburg S.J. (2012). Pathology Supported Genetic Testing and Treatment of Cardiovascular Disease in Middle Age for Prevention of Alzheimer’s Disease. Metab. Brain Dis..

[12] Mahley R.W. (2016). Apolipoprotein E: From Cardiovascular Disease to Neurodegenerative Disorders. J. Mol. Med..

[13] Ray M., Ruan J., Zhang W. (2008). Variations in the Transcriptome of Alzheimer’s Disease Reveal Molecular Networks Involved in Cardiovascular Diseases. Genome Biol..

[14] Wang K., Lu Y., Morrow D.F., Xiao D., Xu C., The Alzheimer’s Disease Neuroimaging Initiative (2022). Associations of ARHGAP26 Polymorphisms with Alzheimer’s Disease and Cardiovascular Disease. J. Mol. Neurosci..

[15] Belloy M.E., Napolioni V., Greicius M.D. (2019). A Quarter Century of APOE and Alzheimer’s Disease: Progress to Date and the Path Forward. Neuron.

[16] Xu C., Garcia D., Lu Y., Ozuna K., Adjeroh D.A., Wang K., on behalf of the Alzheimer’s Disease Neuroimaging Initiative (2021). Levels of Angiotensin-Converting Enzyme and Apolipoproteins Are Associated with Alzheimer’s Disease and Cardiovascular Diseases. Cells.

[17] Broce I.J., Tan C.H., Fan C.C., Jansen I., Savage J.E., Witoelar A., Wen N., Hess C.P., Dillon W.P., Glastonbury C.M. (2019). Dissecting the Genetic Relationship between Cardiovascular Risk Factors and Alzheimer’s Disease. Acta. Neuropathol..

[18] Bessi V., Balestrini J., Bagnoli S., Mazzeo S., Giacomucci G., Padiglioni S., Piaceri I., Carraro M., Ferrari C., Bracco L. (2020). Influence of ApoE Genotype and Clock T3111C Interaction with Cardiovascular Risk Factors on the Progression to Alzheimer’s Disease in Subjective Cognitive Decline and Mild Cognitive Impairment Patients. J. Pers. Med..

[19] Sharma V.K., Mehta V., Singh T.G. (2020). Alzheimer’s Disorder: Epigenetic Connection and Associated Risk Factors. Curr. Neuropharmacol..

[20] Jammeh E., Zhao P., Carroll C., Pearson S., Ifeachor E. Identification of Blood Biomarkers for Use in Point of Care Diagnosis Tool for Alzheimer’s Disease. Proceedings of the 2016 38th Annual International Conference of the IEEE Engineering in Medicine and Biology Society (EMBC).

[21] Amatruda J.G., Estrella M.M., Garg A.X., Thiessen-Philbrook H., McArthur E., Coca S.G., Parikh C.R., Shlipak M.G., TRIBE-AKI Consortium (2021). Urine Alpha-1-Microglobulin Levels and Acute Kidney Injury, Mortality, and Cardiovascular Events Following Cardiac Surgery. Am. J. Nephrol..

[22] Huang Y.-M., Ma Y.-H., Gao P.-Y., Wang Z.-B., Huang L.-Y., Hou J.-H., Tan L., Yu J.-T. (2023). Plasma Β2-Microglobulin and Cerebrospinal Fluid Biomarkers of Alzheimer’s Disease Pathology in Cognitively Intact Older Adults: The CABLE Study. Alzheimer's Res. Ther..

[23] Zhang Y., Yang Y., Wang C., Chen W., Chen X., Wu F., He H. (2023). Copper Metabolism-Related Genes in Entorhinal Cortex for Alzheimer’s Disease. Sci. Rep..

[24] Kim Y., Lu S., Ho J.E., Hwang S., Yao C., Huan T., Levy D., Ma J. (2021). Proteins as Mediators of the Association Between Diet Quality and Incident Cardiovascular Disease and All-Cause Mortality: The Framingham Heart Study. JAHA.

[25] Shi F., Sun L., Kaptoge S. (2021). Association of Beta-2-Microglobulin and Cardiovascular Events and Mortality: A Systematic Review and Meta-Analysis. Atherosclerosis.

[26] Chen F., Liu J., Li F.-Q., Wang S.-S., Zhang Y.-Y., Lu Y.-Y., Hu F.-F., Yao R.-Q. (2023). Β2-Microglobulin Exacerbates Neuroinflammation, Brain Damage, and Cognitive Impairment after Stroke in Rats. Neural. Regen. Res..

[27] Bao X., Borné Y., Johnson L., Muhammad I.F., Persson M., Niu K., Engström G. (2018). Comparing the Inflammatory Profiles for Incidence of Diabetes Mellitus and Cardiovascular Diseases: A Prospective Study Exploring the ‘Common Soil’ Hypothesis. Cardiovasc. Diabetol..

[28] Hertle E., Van Greevenbroek M.M., Arts I.C., Van Der Kallen C.J., Geijselaers S.L., Feskens E.J., Jansen E.H., Schalkwijk C.G., Stehouwer C.D. (2014). Distinct Associations of Complement C3a and Its Precursor C3 with Atherosclerosis and Cardiovascular Disease: The CODAM Study. Thromb. Haemost..

[29] Garcia-Arguinzonis M., Diaz-Riera E., Peña E., Escate R., Juan-Babot O., Mata P., Badimon L., Padro T. (2021). Alternative C3 Complement System: Lipids and Atherosclerosis. IJMS.

[30] Sacks F.M., Furtado J.D., Jensen M.K. (2022). Protein-Based HDL Subspecies: Rationale and Association with Cardiovascular Disease, Diabetes, Stroke, and Dementia. Biochim. Biophys. Acta Mol. Cell Biol. Lipids.

[31] Hu W.T., Watts K.D., Tailor P., Nguyen T.P., Howell J.C., Lee R.C., Seyfried N.T., Gearing M., Hales C.M., Levey A.I. (2016). CSF Complement 3 and Factor H Are Staging Biomarkers in Alzheimer’s Disease. Acta Neuropathol. Commun..

[32] Bonham L.W., Desikan R.S., Yokoyama J.S. (2016). The Relationship between Complement Factor C3, APOE Ε4, Amyloid and Tau in Alzheimer’s Disease. Acta Neuropathol. Commun..

[33] Wang T., Wang X., Yao Y., Zhao C., Yang C., Han Y., Cai Y. (2022). Association of Plasma Apolipoproteins and Levels of Inflammation-Related Factors with Different Stages of Alzheimer’s Disease: A Cross-Sectional Study. BMJ Open.

[34] Simonsen A.H., Hagnelius N.-O., Waldemar G., Nilsson T.K., McGuire J. (2012). Protein Markers for the Differential Diagnosis of Vascular Dementia and Alzheimer’s Disease. Int. J. Proteom..

[35] Mathews P.M., Levy E. (2016). Cystatin C in Aging and in Alzheimer’s Disease. Ageing Res. Rev..

[36] McNicholas K., François M., Liu J.-W., Doecke J.D., Hecker J., Faunt J., Maddison J., Johns S., Pukala T.L., Rush R.A. (2022). Salivary Inflammatory Biomarkers Are Predictive of Mild Cognitive Impairment and Alzheimer’s Disease in a Feasibility Study. Front. Aging Neurosci..

[37] Gevorgyan M.M., Voronina N.P., Goncharova N.V., Kozaruk T.V., Russkikh G.S., Bogdanova L.A., Korolenko T.A. (2017). Cystatin C as a Marker of Progressing Cardiovascular Events during Coronary Heart Disease. Bull Exp. Biol. Med..

[38] Wu H., Du Q., Dai Q., Ge J., Cheng X. (2018). Cysteine Protease Cathepsins in Atherosclerotic Cardiovascular Diseases. J. Atheroscler. Thromb..

[39] Einwoegerer C.F., Domingueti C.P. (2018). Association Between Increased Levels of Cystatin C and the Development of Cardiovascular Events or Mortality: A Systematic Review and Meta-Analysis. Arq. Bras. Cardiol..

[40] West M., Kirby A., Stewart R.A., Blankenberg S., Sullivan D., White H.D., Hunt D., Marschner I., Janus E., Kritharides L. (2022). Circulating Cystatin C Is an Independent Risk Marker for Cardiovascular Outcomes, Development of Renal Impairment, and Long-Term Mortality in Patients With Stable Coronary Heart Disease: The LIPID Study. JAHA.

[41] Van Der Laan S.W., Fall T., Soumaré A., Teumer A., Sedaghat S., Baumert J., Zabaneh D., Van Setten J., Isgum I., Galesloot T.E. (2016). Cystatin C and Cardiovascular Disease. J. Am. Coll. Cardiol..

[42] Carlsson A.C., Jansson J.-H., Söderberg S., Ruge T., Larsson A., Ärnlöv J. (2018). Levels of Soluble Tumor Necrosis Factor Receptor 1 and 2, Gender, and Risk of Myocardial Infarction in Northern Sweden. Atherosclerosis.

[43] Ain Q.U., Sarfraz M., Prasesti G.K., Dewi T.I., Kurniati N.F. (2021). Confounders in Identification and Analysis of Inflammatory Biomarkers in Cardiovascular Diseases. Biomolecules.

[44] Ajoolabady A., Bi Y., McClements D.J., Lip G.Y.H., Richardson D.R., Reiter R.J., Klionsky D.J., Ren J. (2022). Melatonin-Based Therapeutics for Atherosclerotic Lesions and beyond: Focusing on Macrophage Mitophagy. Pharmacol. Res..

[45] Apte R.S., Chen D.S., Ferrara N. (2019). VEGF in Signaling and Disease: Beyond Discovery and Development. Cell.

[46] Mehta A.K., Gracias D.T., Croft M. (2018). TNF Activity and T Cells. Cytokine.

[47] Zhao M., Cribbs D.H., Anderson A.J., Cummings B.J., Su J.H., Wasserman A.J., Cotman C.W. (2003). The Induction of the TNF Death Domain Signaling Pathway in Alzheimer’s Disease Brain. Neurochem. Res..

[48] Zhao A., Li Y., Deng Y. (2020). TNF Receptors Are Associated with Tau Pathology and Conversion to Alzheimer’s Dementia in Subjects with Mild Cognitive Impairment. Neurosci. Lett..

[49] Ortí-Casañ N., Wu Y., Naudé P.J.W., De Deyn P.P., Zuhorn I.S., Eisel U.L.M. (2019). Targeting TNFR2 as a Novel Therapeutic Strategy for Alzheimer’s Disease. Front. Neurosci..

[50] Pillai J.A., Bebek G., Khrestian M., Bena J., Bergmann C.C., Bush W.S., Leverenz J.B., Bekris L.M. (2021). TNFRSF1B Gene Variants and Related Soluble TNFR2 Levels Impact Resilience in Alzheimer’s Disease. Front. Aging Neurosci..

[51] Ortí-Casañ N., Zuhorn I.S., Naudé P.J.W., De Deyn P.P., Van Schaik P.E.M., Wajant H., Eisel U.L.M. (2022). A TNF Receptor 2 Agonist Ameliorates Neuropathology and Improves Cognition in an Alzheimer’s Disease Mouse Model. Proc. Natl. Acad. Sci. USA.

[52] Ortí-Casañ N., Wajant H., Kuiperij H.B., Hooijsma A., Tromp L., Poortman I.L., Tadema N., De Lange J.H.E., Verbeek M.M., De Deyn P.P. (2023). Activation of TNF Receptor 2 Improves Synaptic Plasticity and Enhances Amyloid-β Clearance in an Alzheimer’s Disease Mouse Model with Humanized TNF Receptor 2. JAD.

[53] Steubl D., Buzkova P., Ix J.H., Devarajan P., Bennett M.R., Chaves P.H.M., Shlipak M.G., Bansal N., Sarnak M.J., Garimella P.S. (2020). Association of Serum and Urinary Uromodulin and Their Correlates in Older Adults—The Cardiovascular Health Study. Nephrology.

[54] Thielemans R., Speeckaert R., Delrue C., De Bruyne S., Oyaert M., Speeckaert M.M. (2023). Unveiling the Hidden Power of Uromodulin: A Promising Potential Biomarker for Kidney Diseases. Diagnostics.

[55] Devuyst O., Olinger E., Rampoldi L. (2017). Uromodulin: From Physiology to Rare and Complex Kidney Disorders. Nat. Rev. Nephrol..

[56] Steubl D., Schneider M.P., Meiselbach H., Nadal J., Schmid M.C., Saritas T., Krane V., Sommerer C., Baid-Agrawal S., Voelkl J. (2020). Association of Serum Uromodulin with Death, Cardiovascular Events, and Kidney Failure in CKD. CJASN.

[57] Araújo D.C., Veloso A.A., Gomes K.B., De Souza L.C., Ziviani N., Caramelli P. (2022). A Novel Panel of Plasma Proteins Predicts Progression in Prodromal Alzheimer’s Disease. JAD.

[58] Jack C.R., Bennett D.A., Blennow K., Carrillo M.C., Dunn B., Haeberlein S.B., Holtzman D.M., Jagust W., Jessen F., Karlawish J. (2018). NIA-AA Research Framework: Toward a Biological Definition of Alzheimer’s Disease. Alzheimer’s Dement..

[59] Ahmed R.M., Paterson R.W., Warren J.D., Zetterberg H., O’Brien J.T., Fox N.C., Halliday G.M., Schott J.M. (2014). Biomarkers in Dementia: Clinical Utility and New Directions. J. Neurol. Neurosurg. Psychiatry.

[60] Khoury R., Patel K., Gold J., Hinds S., Grossberg G.T. (2017). Recent Progress in the Pharmacotherapy of Alzheimer’s Disease. Drugs Aging.

[61] Buchhave P. (2012). Cerebrospinal Fluid Levels Ofβ-Amyloid 1-42, but Not of Tau, Are Fully Changed Already 5 to 10 Years Before the Onset of Alzheimer Dementia. Arch. Gen. Psychiatry.

[62] Villemagne V.L., Burnham S., Bourgeat P., Brown B., Ellis K.A., Salvado O., Szoeke C., Macaulay S.L., Martins R., Maruff P. (2013). Amyloid β Deposition, Neurodegeneration, and Cognitive Decline in Sporadic Alzheimer’s Disease: A Prospective Cohort Study. Lancet Neurol..

[63] Jansen W.J., Ossenkoppele R., Knol D.L., Tijms B.M., Scheltens P., Verhey F.R.J., Visser P.J., Aalten P., Aarsland D., Alcolea D. (2015). Prevalence of Cerebral Amyloid Pathology in Persons Without Dementia: A Meta-Analysis. JAMA.

[64] Tibolla G., Norata G.D., Meda C., Arnaboldi L., Uboldi P., Piazza F., Ferrarese C., Corsini A., Maggi A., Vegeto E. (2010). Increased Atherosclerosis and Vascular Inflammation in APP Transgenic Mice with Apolipoprotein E Deficiency. Atherosclerosis.

[65] Austin S.A., Sens M.A., Combs C.K. (2009). Amyloid Precursor Protein Mediates a Tyrosine Kinase-Dependent Activation Response in Endothelial Cells. J. Neurosci..

[66] De Meyer G.R.Y., De Cleen D.M.M., Cooper S., Knaapen M.W.M., Jans D.M., Martinet W., Herman A.G., Bult H., Kockx M.M. (2002). Platelet Phagocytosis and Processing of β-Amyloid Precursor Protein as a Mechanism of Macrophage Activation in Atherosclerosis. Circ. Res..

[67] Guo L.-H., Alexopoulos P., Wagenpfeil S., Kurz A., Perneczky R. (2013). Plasma Proteomics for the Identification of Alzheimer Disease. Alzheimer Dis. Assoc. Disord..

[68] Aggarwal V., Kosian S. (2011). Feature Selection and Dimension Reduction Techniques in SAS.

[69] Muthén B., Kaplan D. (1985). A Comparison of Some Methodologies for the Factor Analysis of Non-normal Likert Variables. Br. J. Math. Stat..

